# Supplementary material for: Are carbon emissions trading and green financial instruments synergistic? -Comprehensive quantitative research based on content analysis
Source: PLoS One. 2024 Mar 7;19(3):e0298601. doi: 10.1371/journal.pone.0298601 (PMC10919601; doi:10.1371/journal.pone.0298601)
Supplement: S1 Data — (ZIP) [file pone.0298601.s001.zip › Data/地级市Z_F工作报告.zip!/║┌┴·╜¡/╞▀╠¿║╙╩╨/└┤╫╘╣1⁄2╓┌║┼í╛┬φ┐╦╩2╛▌═°í┐_2014.docx]

各位代表：

　　现在，我代表市人民政府向大会作工作报告，请予审议，并请各位列席人员提出意见。

　　一、2013年工作回顾

　　2013 年是我市近十年来经济发展最为艰难的一年。面对国内煤焦市场持续低迷、宏观政策趋紧与我市产业结构单一等不利因素叠加冲击，在省委、省政府和市委的坚强领导下，在市人大和市政协的全力支持下，我们凝神聚力、负重前行、扎实工作，积极应对经济下行的巨大压力，在攻坚克难中实现了民生持续改善、社会安定和谐、各项事业稳步发展。

　　经济在困境中逐步企稳。虽然经济总体处于低位运行，但通过努力消化不利因素，主要经济指标止降趋稳。预计地区生产总值实现241 亿元，下降14.5%；公共财政预算收入18.9 亿元，下降13.2%。固定资产投资实现29%的快速增长，收窄了经济下行速度，发展后劲明显增强。在财税收入大幅下降情况下，通过加大对上争取和调节支出结构，保证了全市重点项目、民生工程和社会保障等重大支出，教育、卫生、社保等民生支出占财政总支出比重上升2.5 个百分点，并且在未增加政府性债务的同时，全年偿还贷款9.8 亿元。多渠道增加城乡居民收入，按照公务员工资补贴标准上限提高了工资水平，人均月工资提高130 元；城市低保标准由每月310 元提高到380 元。城镇居民人均可支配收入达到18559 元，增长4.5%；农民人均纯收入达到9080 元，增长10%。内外贸易活跃，社会消费品零售总额实现80 亿元，增长8%；外贸加工企业实现进出口额2400 万美元，增长141%；物价总体稳定，CPI 累计上涨2.2%。

　　转型发展步伐加快。牢牢抓住转型发展中心任务，推进重点产业项目80 个，比上年增加27 个，总投资157 亿元，增长27%，其中非煤项目占92.5%、投资额占87%。宝泰隆和龙盛达两个煤焦油深加工、金泰环己酮己二酸、德彩光电、嘉寓门窗、光明玻璃等一批大项目建成投产，达产后每年可新增产值35.6 亿元。全年产业项目完成投资142 亿元，占全社会固定资产投资67.3%，占比位居全省前茅。市级财政投入3 亿元支持产业园区基础设施建设，园区承载能力增强，省市级重点园区引入新项目33 个。产业结构明显变化，二产比重下降11.4 个百分点，一产和三产分别上升3.0 和8.4 个百分点，三次产业比重为13.2：46.8：40.0。规模上工业增加值煤焦电化比重比上年下降1 个百分点，非煤工业比重提高到10%。通过创新组织动员、激励约束和项目生成机制，招商引资取得新突破，第一轮专业招商共引进项目30 个，总投资42 亿元。

　　“三农”工作扎实推进。农业战胜春涝、低温等自然灾害，粮食产量实现“十连增”。规模化种植、养殖水平提高，全市土地流转面积104 万亩，特色经济作物面积达到21 万亩；新增标准化畜牧养殖场22 个，新建4 个畜牧科技产业园区。农村合作组织进一步壮大，农民合作社达到366 个。农业产业化程度稳步提升，市级以上龙头企业带动基地农户6 万户，标准化种植80 万亩，拉动户均增收1700 元。转移农村劳动力9 万人，实现劳务收入8.4 亿元。城乡一体化进程加快，累计投入新农村建设资金3.9 亿元，开展了2 个省级百镇试点和5 个小城镇试点建设工作，万宝村、红光村实现整村改造搬迁。农村各项改革逐步深入，制定出台《强镇扩权财政优惠政策》等改革实施办法，市直各部门向乡镇下放行政权力17 项，有效激活镇域经济发展潜力，强镇扩权改革初见成效。

　　民生和社会事业取得新进展。研究制定了《重点民生工程三年行动计划》，着力推进7 个方面43 项重点工程，70%的民生项目启动，超过三分之一的项目取得阶段性成果。在省政府支持下，城市供水工程获得批复，基本完成开工准备。实施农村饮水安全工程，解决了3.2 万人饮水困难。安居工程加快推进。投入5.3 亿财政资金加大棚户区等保障性住房建设。完成22626 套煤矿棚改房建设，新开工10499 套，严格棚改复核审查，纠正不合理安置面积1 万多平方米，挽回直接经济损失1944 万元，节约房源3000 余套，保证了公平分配，21268 户居民告别棚户区；新建城市棚改房、廉租房、公租房、经济适用房5884 套；改造农村泥草房、危房3801 户；完成5 个小区57 栋住宅楼“暖房子”工程。社会保障能力稳步提升。社会保险参保人员净增3.2 万人，个体养老保险和城镇职工养老保险每月人均提高230 元，城镇职工医保、城镇居民医保和大病统筹报销年度累计最高支付额分别提高至10 万元、6 万元和23 万元；加强对困难群体的救助，建立生活困难失能老人护理补贴制度；加大就业帮扶力度，新增就业1.2 万人，再就业8509 人。社会事业全面发展。市人民医院外科楼等14个卫生基础设施项目进展顺利。新一中一期工程竣工，新建改造2 所寄宿制学校、9 所公办幼儿园，66 所学校进入省级标准化学校；课改工作走在全省前列，获得全国“十大区域课改样板市”称号；高考录取率达到87.7%，比上年提高2.7 个百分点。文体事业加快发展，“文化三馆”免费向市民开放，群众翘首以盼的体育会展中心投入使用；竞技体育取得新成绩，短道速滑项目获省级以上奖牌65 枚。

　　城市建设管理水平提升。完成依宝公路东出口和大同街东延一期等道路工程，改造同仁中巷等13 条2.3 万平方米巷道。新建、维修、改造供水管网23 公里、供热管网11 公里，基本完成第二汇水区雨污分流管网续建工程。第二污水处理厂加快建设，医疗废物处置中心启动运行。万宝110KV 输变电工程、长兴35KV 增容改造工程顺利推进。加大违建清理、小锅炉淘汰等专项整治力度，开展了以污水、垃圾为重点的桃山湖污染治理行动，完成主要污染物年度减排目标。完善城市出入口景观建设，实施了东进街、北岸新城等绿化工程，建立倭肯河省级湿地自然保护区和桃山湖国家湿地公园，建成区绿化覆盖率达到43.7%。全面完成“十项整治”工作，深入实施“六化一考”城市管理机制，市容市貌整体改善，成功晋升为“国家园林城市”。

　　社会保持和谐稳定。全力应对多年不遇暴雪灾害，各级各部门迅速行动、昼夜奋战，尽最大努力减少了灾害损失和影响。全面推进安全监管体制机制改革，落实企业主体责任，健全属地管理责任体系，全方位、全覆盖、高频次开展隐患排查和安全大检查大整顿，危化、交通、消防等领域安全生产形势稳定，未发生3 人以上事故。食品安全、物价稳控、打非治违工作得力有效。研究提出煤矿集团化整治整合思路，72 处煤矿通过省审核。出台系列地方煤矿安全监管规范性文件，严格落实24小时旁站式监管，煤矿事故起数和死亡人数分别下降41.7%、38.5%。加大矛盾纠纷排查化解力度，研究出台相关政策，投入1 亿多元解决了一批历史遗留信访积案，妥善化解369 件信访疑难问题，案件化解率达72.3%，近千人息访。“平安七台河”创建深入开展，织密社会治安动态防控网，社会治安持续好转。

　　政府自身建设不断加强。进一步清理行政审批事项，全年共取消、下放、合并86 项，占原审批事项的31.2%。全部免除市级行政事业性收费，开辟重大产业项目审批“绿色通道”，实行联合审批、“保姆式”服务，行政效能进一步提高。出台《鼓励非公经济发展促进全民创业的若干意见》，设立中小微企业发展种子基金。组织大型银企对接活动10 余次，为企业解决资金60 多亿元。高度关注社情民意，开通政府网“民声之窗”专栏，扎实开展“联包助帮”和“三办”活动，定期召开民情恳谈会、企业家恳谈会，积极解决企业和群众实际困难。认真执行市人大及其常委会决议决定，自觉接受人大法律监督和政协民主监督，按时办结人大代表建议40 件，政协委员提案185 件。认真贯彻落实中央八项规定，深入整改“六多六少”问题，加大作风建设监督检查力度，问责和曝光了一批典型案件。树立过紧日子思想，严格控制公用经费，公务用车和公务接待费用分别下降24.8%、32.6%。全面落实党风廉政建设责任制，强化行政监察和审计监督，反腐倡廉建设进一步加强。

　　这些成绩的取得，是各行、各界、各部门通力协作，辛勤付出的结果，是全市人民共同努力的结果。在此，我代表市人民政府对全市人民和各级各界的支持与帮助表示衷心感谢！

　　过去的一年，虽然我们有效落实了一些打基础、利长远、促发展、惠民生的实事要事，但与全市人民的要求和期盼还存在较大差距。一是转型发展的长期性与现实要求的紧迫性矛盾更加突出，支撑转型的产业项目发展缓慢、成长性不高，单一产业结构没有根本改变。二是城市基础设施不完善，部分供水供热供气管网老化，一些居民区不能全天供水，欣源小区供气不稳定，有的地方供热不达标；公交场站、市民活动场所等公共服务设施满足不了群众需求，机械化清雪设备不足，应急保障能力不强。三是环境污染问题仍很突出，煤烟型大气污染严重，多次出现雾霾天气，部分企业环境保护责任意识不强。四是对困难群体的救助帮扶还不到位，一些低收入家庭生活比较困难。五是信访矛盾突出，尤其是多年积压疑难信访案件未能有效解决，应对社会矛盾凸显期的能力不足。六是安全生产隐患较多，部分安全标准、措施形同虚设，煤矿整治整合尚未取得实质性进展，危险化学品等领域安全保障能力薄弱，属地和行业安全监管不到位等问题依然突出。七是部分领导干部仍然存在“四风”问题，一些干部萎靡不振、懒惰麻木，纪律松弛、我行我素，工作避重就轻、挑肥拣瘦，不能很好履职尽责。这些问题，必须高度重视并认真加以解决。

　　二、2014年工作安排

　　2014 年是深入贯彻落实党的十八届三中全会精神、全面深化改革的第一年，是我市实现“十二五”规划目标、推进城市转型发展的关键之年。当前及今后一定时期，全国煤炭市场供大于求的状况难以改变，结构性过剩的态势仍将延续，我市经济下行、财政短收的压力依然存在，改革发展的任务繁重而艰巨。但也要看到，我们正面临着难得的发展机遇。虽然世界经济仍然存在诸多不确定性，但我国经济发展长期向好的基本面没有变；全面深化改革将激发新的发展动力，国家坚持稳中求进总基调，继续实施积极的财政政策和稳健的货币政策，将为经济发展创造稳定的外部环境。我省“两大平原”现代农业综合配套改革、对俄沿边开发开放、大小兴安岭生态功能区、老工业基地振兴等“五大规划”上升为国家战略，将极大地释放经济发展潜能。特别是实施扶持资源型城市可持续发展规划，有利于我们加快推动七台河转型发展。我市经过三年产业项目建设，一批煤炭循环经济大项目、转型替代产业好项目相继建成投产，城市矿产示范基地、煤化工产业园、经济开发区等产业园区承载能力不断增强。城市发展的软硬环境全面改善，全市干部群众抓项目促发展的意识更加强烈，这些都为今后发展奠定了基础，积蓄了后劲。

　　做好2014 年工作，我们既要勇于正视和积极破解存在的困难和问题，又要充分认识积极因素和把握有利条件，坚定信心、攻坚克难，科学施策、狠抓落实。全年工作的总体要求是：以邓小平理论、“三个代表”重要思想和科学发展观为指导，全面贯彻落实党中央、国务院系列部署和要求，落实省委十一届四次全会、省委经济工作会议、市委八届四次全会精神，以全面深化改革为统领，以稳中求进为基调，以加快转型为中心，坚持依托煤、延伸煤、不唯煤、超越煤的发展思路，全力以赴打基础、稳增长、调结构、促改革、惠民生、保稳定，努力开创“推动四个发展，建设幸福之城”新局面。

　　主要预期目标：地区生产总值增长6%左右，规模以上工业增加值增长8%以上；公共财政预算收入增长5%以上；全社会固定资产投资增长20%以上，其中产业投资比重达到60%以上；消费品零售总额增长10%左右；外贸进出口总额增长8%以上；城镇居民人均可支配收入增长7%以上，农民人均纯收入增长8%以上。

　　全年重点抓好六个方面工作，落实36 件惠民实事。

　　（一）全面深化改革，增强发展活力和动力

　　全面落实十八届三中全会精神，突出公共资源市场化改革，破除影响发展体制机制障碍，增强经济发展活力和动力。加快推进市场化进程。充分发挥市场在资源配置中的决定性作用，对土地出让等政府性资源实行公开招标、拍卖。探索利用BOT、BT、特许经营等市场化路径解决城市供热、供气、给排水和垃圾处理等问题。清理妨碍市场公平竞争的不合理政策，健全社会诚信体系，充分发挥市场竞争作用。深化行政管理体制改革。进一步优化政府机构设置、职能配置，建立高效运行机制。组建卫生计划生育委员会，理顺食品药品监督管理局职能。减少行政管理层级，撤销街道管理机构，实现新型社区全覆盖。进一步简政放权，深化行政审批制度改革，最大限度减少审批事项，全面优化审批流程,实行部门联动，畅通行政审批服务环节,创建中介服务超市,推行网上审批。深化招投标改革，建立公共资源交易中心，制定统一的政策法规，组建统一的管理机构，构建统一的交易平台，实行统一的专家库，形成统一的监管体系。严格政府部门绩效管理，完善区县考核评价体系，增加产业投资、环境保护、安全生产、劳动就业等指标权重。深化城市投融资体制改革。激活政府资源资本属性，盘活城市存量资产，广泛吸纳社会资本，组建国有资产管理（集团）公司，推动政府投资、融资、管理、偿债等权责一体化管理，使城市建设、产业发展、园区建设更多地依靠市场力量推动。深化财税体制改革。进一步完善市区分税制改革政策和配套制度，理顺收入关系，厘清支出责任。深化预算管理改革，全面编制公共财政预算、政府性基金预算、国有资本经营预算、社会保险基金预算，细化部门、政府采购等预算编制，形成全面规范、公开透明的预算管理制度，加强财政资金审计监督，确保财政资金安全规范运行。完善政府性债务管理，建立政府债务管理及风险预警机制，控制政府债务规模。取消一般性公务车，推动公务用车制度改革。深化教育、卫生等社会体制改革。建立教师交流轮岗制度和定期考核制度，实行能进能出的动态用人机制。改革初高中录取方式，促进教育均衡、公平发展。推进公立医院改革，建立现代医院管理制度，取消市直3 家公立医院药品加成，解决以药补医问题。允许医师多点执业。推动城镇居民和职工医保、新农合“三保合一”改革，转变医保支付方式。落实“单独两孩”政策。继续推进和完善强镇扩权、司法体制、事业单位、煤炭税收征管体制等改革，确保改革任务落到实处。

　　（二）全力推进产业项目建设，加快构建支撑转型产业体系

　　坚持做大增量与优化存量并进，重点推进千万元以上产业项目62 个，总投资181 亿元，年度投资44 亿元，力争30 个项目建成投产。其中，非煤产业项目57 个，总投资138 亿元，年度投资36 亿元。大力发展新型煤化工产业。支持焦炭企业适应市场之变，开发新产品、延伸产业链、谋划新出路，转冶金焦为化工焦。积极促进宝泰隆和龙盛达两个煤焦油深加工、金泰环己酮己二酸等项目投产达效，加快伊普润滑油、龙盛达煤焦油二期项目建设，争取宝泰隆30 万吨芳烃、隆鹏2 亿立方米LNG 项目开工。鼓励支持宝泰隆、七煤等煤企、化企联合协作，开发煤焦油、甲醇下游筑路沥青、醋酸、二甲醚等精细煤化工项目。加快煤矿探转采步伐，推动七煤公司七峰一井、宝泰隆等新矿井建设，支持七煤公司争取后备资源，开发域外资源。坚持一个矿区一个开发主体，加快地方煤矿整治整合，实现资产、资源联结的集团化。壮大非煤替代产业集群。发挥绿色食品产业发展潜力，加快正邦集团生猪产业化、天泰公司玉米食品、佐源集团菊苣糖、金瀛公司纳豆等项目建设，壮大绿色食品产业。巩固销地市场优势，抓好勃农宽窄行水稻插秧机、泰安抑爆隔爆装置、鑫盛机械矿用产品等机械制造项目，壮大机械制造产业。依托石墨、煤矿伴生资源、生物质资源，开发建设精纯石墨、节能建筑材料、清洁型煤等精深加工和综合利用项目，抓好佳美集团无机纤维、晴龙公司脱硝催化剂等高科技项目建设，培育壮大新兴产业。推进工业存量升级。支持双叶公司研发新产品，扩大市场份额，带动木制品园区企业专业化分工和集群化发展。发挥宝泰隆循环经济研究院示范带动作用，积极引进和储备高端人才，加强企业创新能力建设。落实省政府“工业新十七条”，鼓励企业联合做大做强，研究煤矿开采企业转型发展支持政策，继续开展银企对接，促进企业稳定发展。扩大种子基金至2000 万元规模，发挥放大效应，扶持中小微企业发展。增强第三产业发展活力。加快建设城市综合体，满足和扩大不同层次消费需求。大力发展各类专业市场，建设完善万通物流项目，加强与大连港、铁路内陆港及边境口岸联系，促进商贸物流业集群发展。推进西大圈和石龙山国家森林公园综合开发，谋划开发桃山湖风景区，大力发展休闲养生度假、森林避暑等旅游项目，鼓励体育健身、休闲娱乐等产业发展。加快发展金融、保险、信息、技术等现代服务业。提升园区建设和管理水平。继续推进省市级重点园区水电路热等基础设施建设，打通经济开发区、金沙食品园区对外联络通道，建设经济开发区孵化器、煤化工园区化工路与河堤路、再生园区固废处理中心等项目，提升园区承载能力。因地制宜规划建设县区产业园，加快项目集中摆放和企业集群发展。进一步推行“保姆式”服务，加强入驻园区项目监管，对占而不建、久拖不投的项目实行退出机制，促进企业守法依规生产经营。提高产业项目招商成效。深入推进第二轮招商工作。创新招商方式，组建由市领导牵头的专业招商组，强化专业招商和“双百”招商，严格落实各级、各部门领导招商责任制，重奖重用有功人员。打破市内区域界限，统筹集约利用土地资源，扩大招商项目落地空间。研究招商引资政策，严格兑现产业园区招商、产业项目建设等系列政策。

　　（三）扎实做好“三农”工作，建设美丽幸福乡村

　　坚持以城镇化、产业化为带动，抓住“两大平原”现代农业综合配套改革机遇，创新农业生产经营机制，大力发展特色农业，提升小城镇、新农村建设水平，促进城乡一体化发展。大力发展特色农业。加快标准粮田建设，实施桃山水库灌区中低产田改造工程，扩大水稻种植面积4 万亩以上。突出抓好特色经济作物种植，加快白瓜、有机杂粮、菊苣等种植基地建设，全市特色农业生产专业村达到35 个，特色经济作物种植面积稳定在20 万亩以上。大力开发林下经济，推进黑木耳、香菇等食用菌标准化生产基地建设，打造森林食品品牌。做大做强特色果蔬产业，发展城郊设施蔬菜栽培，力争每个建制镇新建一个蔬菜小区，重点发展提早延晚时令瓜果蔬菜，果蔬生产基地稳定在5000 亩。推广绿色生态养殖技术，扶持建设一批具有区域特色的专业养殖小区，新增规模化畜牧小区10 个、规模大户15 个，提高畜牧业标准化、规模化生产能力。壮大绿色食品产业。发挥金沙食品产业园区、勃利食品产业园区等园区承载作用，大力引进和扶持绿色食品加工龙头企业，促进大森林、大兴牧业等龙头企业做大做强。加强绿特色品牌创建，创新农产品交易方式，鼓励通过农超对接、网络销售等形式开拓全国绿色食品市场，绿色食品产业销售收入实现25 亿元，农产品出口额700万美元。构建新型农业经营体系。通过以奖代投等方式，重点扶持发展专业大户、家庭农场、农民合作社和农业产业化企业，建立财政资金扶持新型农业经营主体稳定增长机制。开展农民土地承包经营权确权登记试点工作，建立农村土地流转交易平台，引导农村土地经营权有序流转，推动土地适度规模经营，土地规模经营面积达到80 万亩。大力培育新型职业农民，加大富余劳动力培训和转移力度，力争实现劳务收入8.5 亿元以上。加快城乡一体化进程。合理布局村镇建设空间结构，完善各具特色的村镇规划建设，促进人口向城镇和中心村集中，产业向城镇集聚。推进万宝河镇、双河镇等7 个小城镇试点建设，完成万宝村等4 个整村改造和红鲜村等3 个整村搬迁。继续推动村企共建，多渠道筹集资金，加强村镇道路、文化广场、农家书屋等基础设施和公共服务设施建设，完善农村垃圾处理、卫生改厕等环保设施，加强村镇绿化、美化，加快农村泥草房、危房改造，全面改善农村生产条件和人居环境。

　　（四）加强城市基础设施建设，打造山水宜居乐业城市以实现城市的绿色承载为理念，强化城市规划、建设、经营、管理，提升山水宜居乐业城市品质。拓展城市发展空间。⑴规划实施大同街东延二期等区间联络通道，促进茄子河区与桃山区融合发展，增强中心城区与金沙新区、经济开发区的发展联系；改造延伸桃山湖北路，借地借势向东向北拉开城市布局框架。提高基础设施承载能力。(2)新建改造松涛路、景丰路南延等工程，拓宽北岸新城区段七桦路。(3)加快“三供”管网建设，推进中燃集团天然气项目，金沙新区、万宝英伦小镇率先使用天然气。(4)开工建设和改造茄子河变电所、金沙新区变电所，满足电力增长需要。(5)继续推进雨水管网工程，完成第二污水处理厂、污泥处置厂及配套管网建设。(6)开工建设锦江集团生活垃圾焚烧发电项目，实现生活垃圾全部集中减量化、无害化处理。(7)优化城市公共交通基础服务设施，建设北岸新城、金沙新区和大同街东段三处公交调度指挥中心。建设山水宜居家园。(8)抓好学府街东延、大同街东延、文华路、景丰南路绿化升级工程，提升居住小区和单位庭院绿化水平。(9)开工建设月亮湾广场和水上公园。(10)强化大气雾霾治理，制定实施《七台河市清洁空气行动计划》，做好焦化厂电厂脱硫脱硝、小锅炉改造、淘汰黄标车、餐饮业污染防治工作，开展PM2.5 监测发布工作。(11)加强桃山湖水源地污染综合防治，完成东胜村污水泵站及茄子河截污管道续建工程。(12)建立重点流域环境保护“河长制”，完善倭肯河流域污染控制单元和分区管理体系。(13)建设桃山湖国家湿地公园，分期推进倭肯河坝下生态景观工程建设。强化城市经营管理。创新城市建设管理模式，鼓励社会资本参与基础设施投资和运营；通过政府购买服务等方式，提高市政设施维护、城市绿化养护、环卫保洁等市场化程度。做好月亮湾、英伦小镇二期、大同街东延两侧土地收储和开发，加快棚改配套商服用房开发建设。严格控制棚改腾空土地使用，启动土地整理工作。完善动迁管理办法，规范动迁行为，缓解城市动迁、拆迁矛盾。(14)推动“六化一考”向县区延伸，继续推进环境综合整治工作，提高垃圾、冰雪机械化清运能力，严格控制占道经营、违建滥建行为。(15)加快建设数字七台河地理空间框架，有序分步推进智慧城市建设。(16)强化城市综合管理指挥中心服务职能，促进居民参与城市管理。(17)推动物业管理扩面提质，引入服务竞争机制，建立监督考评制度，提高北岸新城等居民新区物业管理水平，形成长效机制。(18)建立“弃管楼”物业管理责任追究机制，实现居民委员会自治化、市场化管理。(19)做好龙煤公司“三供一业”接收后管理运营工作。

　　（五）加快发展社会事业，着力保障和改善民生

　　全面推进实施《重点民生工程三年行动计划》，抓好民生实事落实，促进社会事业全面发展。加快实施城市供水工程。(20)全力做好城市供水工程土地林地征用、移民动迁安置、工程招标工作，与鸡西同步协调开工建设，主体工程与附属工程整体推进。推进保障性住房建设。(21)重点做好已开工保障房收尾工程，严格核查核实，确保公正公平公开及时分配。建成煤矿棚改房10499 套，做到及时安置、连片拆除。完成城市棚改房4594 套、廉租房1800 套、公租房350 套。(22)继续实施“暖房子”工程，改造老旧住宅100 万平方米。积极促进就业创业。(23)落实就业政策，鼓励各类创业带动就业。发挥失业保险促进就业作用，通过发放小额担保贷款、多渠道开发就业岗位等方式，促进劳动力充分就业。突出做好煤矿下岗工人、农民工、“4555”人员的就业指导和扶持工作，动态消除“零就业”家庭。(24)强化劳动监察，切实保障矿工、农民工等劳动者权益，构建和谐劳动关系。增强社会保障能力。(25)促进社会保险由制度全覆盖向人员全覆盖转变，加大保费征缴和管理力度，建立社会保险与相关部门对应业务联查联用机制。(26)加强低保科学管理，严格落实提标政策。(27)按照全市筹资规模1000 万元以上、各区100 万元以上标准，设立城乡困难群众救助帮扶基金，对低收入家庭每人每年一次性发放150 元生活补贴，提高对伤残、重病等特困家庭救助标准。(28)完善现有公办养老服务机构基础设施，加强民办养老机构达标改造和安全监管，扩大居家养老服务覆盖面。(29)提高基层民警补贴和社区工作人员工资。促进社会事业全面发展。(30) 增加教育投入，改善学校教学设施，加强学校内涵建设，加快教育信息化建设步伐，提高教育教学水平。(31)完善职业技师学院发展思路，深化产教融合、校企合作，搭建大学生创业孵化基地。(32)完成新一中建设，配齐配强师资力量，打造优质高中。(33)推进北岸新城、如意家园等新区普惠性幼儿园建设，改扩建市实验幼儿园，提高公办幼儿园比重。(34)统筹市域内医疗资源布局，完成市人民医院外科楼、市中医院收尾工程，开工建设市妇幼保健院，增强中医院自我发展能力。(35)继续完善乡村卫生院所、社区卫生中心、“健康小屋”等基层医疗服务设施，乡镇卫生院至少引进一名具有本科学历的医生。(36)加快文化体育事业发展，大力开展群众文化活动，开工建设市综合文化活动中心，提高体育会展中心使用效能和运营水平。

　　（六）强化综合治理，维护社会和谐稳定

　　推进平安七台河建设，健全公共安全体系，确保人民安居乐业，社会安定有序。加强安全生产工作。树立安全生产红线意识，强化“管行业必须管安全”责任，健全安全生产全程监督机制，加强安全标准化建设，加大隐患排查和安全巡视，严厉打击非法生产行为。突出抓好煤矿安全工作，落实党政同责，强化“四个责任”和落实“矿长保护矿工七条规定”，推进煤矿水患物探和警戒线、禁采线划定工作，坚持24 小时旁站式监管和“五化一加强”、“六防”等各项工作制度，确保煤矿安全生产。提升危险化学品事故应急处置能力，加强“两重点一重大”和化工全过程安全管理。建立覆盖城乡的食品药品、农产品质量监测体系。抓好特种设备、交通运输、非煤矿山、建筑、消防等重点领域的安全工作。积极化解信访矛盾。强化属事属地责任有效统一，变等访为下访和查访，全面摸清苗头性、倾向性和潜在性的矛盾隐患。落实信访包案责任制，完善三级信访终结制度，建立健全信访信息反馈报告制度。进一步畅通群众诉求渠道，探索创新依法处访的新途径，实施信访案件分类办理和倒查机制，制定信访案件溯源追责办法，妥善解决好群众关心的热点和难点问题。加强社会治安综合治理。坚持开展各类严打专项整治活动，统筹部署警力下沉工作，增加辅警数量，提高街面见警率，坚决遏制“两抢一盗”和恶性案件发生。加强政府应急指挥平台建设，完善各类事件应急预案，做好应急物资储备，提高应急处置能力，有效应对各类突发事件，确保人民群众生命财产安全。

　　三、改进政府工作

　　面对发展攻坚的新任务和人民群众的新期待，扎实开展好群众路线教育实践活动，以饱满的精神状态和良好的工作作风，着力打造人民满意的服务型政府。

　　打造促进发展的优良环境。转变政府职能。把握好政府与市场、政府与社会的关系，通过深化行政体制改革，把工作的重点转到创造良好发展环境、提供优质公共服务、维护社会公平正义上来，把该放的权力放开放到位，把该管的事务管住管好。强化服务举措。深入开展“联包助帮”和“三办”活动，充分发挥民声之窗、民情恳谈会、企业家恳谈会等载体平台作用，为加快发展提供更周到、更具体、更优质的服务。整治发展环境。坚决整治查办部门和个人政令执行不畅的“层级梗阻”问题、不负责任的“上推下卸”问题、办事拖拉的“庸懒散软”问题、以权谋私的“吃拿卡要”问题等，下最大气力打造暖人心、留住人、有发展的优良环境。

　　坚持真抓实干的工作作风。创新工作抓落实。探索推行三分之一工作法，领导干部要三分之一时间下基层，解决企业和群众实际困难；三分之一时间抓招商，用心搞好产业项目建设；三分之一时间抓业务，扎实做好基础性工作。提升能力抓落实。形成良好的学习风气，少应酬多学习、少扯皮多思考、少空谈多干事，静下心来读书，凝住神来工作，切实增强抓落实的前瞻性、主动性和针对性。强化责任抓落实。实行重点工作督办通报制，加大对全市安全生产、重点项目建设、重大事项落实等督查督办和督导力度，提高政府执行力和行政效率；严格实行重大问题责任倒查机制，严肃查处重大工作落实中的失职渎职行为。

　　树立清正廉洁的良好形象。始终坚持群众路线，以群众的渴盼作为工作的动力，把群众的满意程度作为衡量工作的标准，坚持力量向一线倾斜、问题在一线解决、干部在一线检验、形象在一线树立，实实在在为群众办实事解难题，打造老百姓贴心的政府。认真贯彻落实中央“八项规定”、国务院“约法三章”，从整改群众反响最强烈的超标接待、违规用车、奢侈浪费等作风问题入手，坚决整治广大人民群众深恶痛绝的形式主义、官僚主义、享乐主义和奢靡之风，构建权责清晰、流程规范、制度管用、预警及时、防控有效的廉政风险防控管理机制，打造一支廉洁干事的干部队伍。切实把权力关进制度的笼子里，用制度管权管事管人，自觉接受人大、政协和人民群众监督，建立重大决策定期通报制度，做到依法行政、规范用权，打造阳光廉洁的政府。

　　保持奋发有为的精神状态。牢记宗旨使命。始终不忘加快发展第一要务，强化为民服务意识，提起干事创业的精气神，带着感情、责任和追求去工作，克服当一天和尚撞一天钟的庸碌无为、萎靡麻木状态。坚定发展信心。充分把握现阶段经济社会发展的规律和特点，牢牢抓住稍纵即逝的发展机遇，不断突破工作新局面，克服畏难不前、瞻前顾后的思想作风。增强开拓意识。时刻保持逢山开路的进取精神，解放思想，创新实践，在招商引资、项目建设、城市管理等重点工作上敢闯敢试，努力探求破解发展瓶颈的思路和办法，克服因循守旧、固步自封的工作习惯。

　　各位代表，新的一年新的愿景，群众有新期待，发展有新要求。重任在肩，时不我待，让我们在省委省政府和市委的正确领导下，在人大和政协的监督支持下，坚定信念不动摇，开拓进取不懈怠，埋头实干不松劲，不断开创建设幸福之城新局面！

《政府工作报告》名词解释

　　“文化三馆”：博物馆、图书馆和群众艺术馆。

　　“六多六少”：我市在查找“四风”方面存在的具体问题，主要是安排部署比较多，检查推动和解决具体问题比较少；抓面上的问题比较多，抓点上的问题比较少；检查先进典型的时候比较多，到困难大、矛盾多的地方比较少；坐在办公室的时间比较多，深入基层面对面实打实解决困难比较少；被动应付工作的比较多，主动想事干事、积极改革创新的比较少；对各种超标问题和不良风气习以为常的比较多，自觉主动整改的比较少。

　　“工业新十七条”：省工信委、发改委、财政厅等十一个部门联合发布《关于促进全省工业经济稳定增长的若干政策措施》,简称“工业新十七条”。

　　“双百”招商：市委市政府于2013 年探索推行的招商新模式，即，在域外聘请100 名各界人士任驻外信息员，派出100 名优秀干部到经济发达地区挂职招商。

　　“河长制”：由各级党政主要负责人担任“河长”，负责辖区内河流的污染治理。“河长制”是从河流水质改善领导督办制、环保问责制所衍生出来的水污染治理制度，目的是为了保证河流在较长的时期内保持良好的生态环境。

　　“三供一业”：供水、供热、供气和物业管理。

　　“4555 人员”：女性年满45 周岁以上、男性年满55 周岁以上的人员。

　　“学校内涵建设”：学校制度、学校文化、学校管理等建设。

　　“健康小屋”：政府主导，由公共卫生机构提供给市民用于体检测量、干预指导、健康宣教的场所。

　　“六化一考”：全市城市管理新模式，包括市场化、规范化、精细化、人性化、群众化、数字化，建立城市管理考评体系。

　　“四个责任”：企业安全生产主体责任、政府监管责任、地方政府属地管理责任和岗位责任。

　　“五化一加强”：全市地方煤矿安全生产方面建设标准化、操作规范化、管理制度化、设备安标化、培训法规化，加强煤矿的监管，落实监管队伍的责任。

　　“六防”：是指地方煤矿防瓦斯、防水害、防煤尘、防火灾、防冒顶、防跑车六项防治隐患措施。

　　“两重点一重大”：政府安监部门重点监管的危险化工工艺、重点监管的危险化学品和重大危险源的监管，简称“两重点一重大”。

　　“联包助帮”和“三办”活动：指市委开展的“联企、包区、助村、帮户”和“农事村办、企事厂办、居民事社区办”活动，是全市各级领导干部转变作风、密切联系群众的重要载体。

　　“八项规定”：2012 年12 月4 日新一届中央领导集体提出的改进调查研究、精简会议活动、精简文件简报、规范出访活动、改进警卫工作、改进新闻报道、严格文稿发表、厉行勤俭节约等八项要求，是我们党转变工作作风，密切联系群众的重要规定。

　　“约法三章”：2013 年3 月全国两会后，李克强总理在与中外记者见面时郑重提出：本届政府任期内，政府性的楼堂馆所一律不得新建，财政供养人员只减不增，公费接待、公费出国、公费购车只减不增。
